# Alpha2-Adrenoblockers Regulate Development of Oxidative Stress and Cognitive Behaviour of Rats under Chronic Acoustic Stress Conditions

**DOI:** 10.3390/ph14060529

**Published:** 2021-06-02

**Authors:** Magdalina Melkonyan, Ashkhen Manukyan, Lilit Hunanyan, Artem Grigoryan, Hayk Harutyunyan, Lilit Sukiasyan, Lusine Danielyan, Konstantin Yenkoyan

**Affiliations:** 1 Department of Medical Chemistry, Yerevan State Medical University after M. Heratsi, Yerevan 0025, Armenia; magda.melkonyan@icloud.com (M.M.); manukyanashkhen@mail.ru (A.M.); h-lilit@live.com (L.H.); 2 Department of Pathophysiology, Yerevan State Medical University after M. Heratsi, Yerevan 0025, Armenia; grigartem@yahoo.com; 3 Laboratory of Neuroscience, Yerevan State Medical University after M. Heratsi, Yerevan 0025, Armenia; hayk@web.am; 4 Laboratory of Morphological Studies, SRS, Yerevan State Medical University after M. Heratsi, Yerevan 0025, Armenia; lilit.sukiasyan@inbox.ru; 5 Department of Clinical Pharmacology, University Hospital of Tuebingen, 72076 Tuebingen, Germany; lusine.danielyan@med.uni-tuebingen.de; 6 Department of Biochemistry, Yerevan State Medical University M. Heratsi, Yerevan 0025, Armenia

**Keywords:** noise, α^2^-adrenoblockers, oxidative stress, malondialdehyde, α-Tocopherol, anxiety-related behavior

## Abstract

Noise is a wide-spread stress factor in modern life produced by urbanization, traffic, and an industrialized environment. Noise stress causes dysfunction and neurotransmission impairment in the central nervous system, as well as changes in hormone levels. In this study, we have examined the level of α-Tocopherol (α-T) and malondialdehyde (MDA) in plasma and the erythrocytes’ membrane (EM), as well as the behavioral characteristics of a noise-induced stress model in rats. In addition, the modulating effect of α^2^-adrenoblockers, beditin, and mesedin on the aforementioned parameters has been investigated. For these purposes, albino male rats were divided into four groups: (1) untreated; (2) noise-exposed, (3) noise-exposed and beditin-treated (2 mg/kg, i.p.), and (4) noise-exposed and mesedin-treated (10 mg/kg, i.p.) animals. Noise-exposed groups were treated with 91dBA noise on 60 days with a daily duration of 8 h. Increased MDA and decreased α-T levels in plasma and EM were observed upon chronic high-level noise exposure. Locomotor and behavioral activity assessed with a Y-maze revealed disorientation and increased anxiety under chronic noise exposure. Prominently, α^2^-adrenoblockers alleviated both behavioral deficits and oxidative stress, providing evidence for the involvement of α^2^-adrenoceptor in the pathophysiology of noise-induced stress.

## 1. Introduction

Noise is an unwanted product of urbanization posing increasing health hazards for humans and animals [1]. It has been proven to be a common cause of hearing loss and may have negative health impacts not only at auditory but also non-auditory level. On the one hand, noise is one of the most common causes of stress [2]. Noise exposure leads to an overactivation of the sympathetic system, resulting in increased levels of noradrenalin, adrenalin, angiotensin-II, and cortisol, which in turn, activates endothelial NADPH oxidase, leading to oxidative stress [3]. Noise-induced stress promotes the generation of free oxygen radicals with the following modification of macromolecules such as nucleic acids, proteins, and particularly fatty acids of phospholipids or lipoproteins, resulting in lipid peroxide formation. In oxidative stress assessment, malondialdehyde (MDA) serves as a marker of lipid peroxidation (LPO)-mediated tissue damage [4,5,6]. This pathological state is associated with progressive free-radical oxidation [7,8], resulting in functional disturbances in various tissues. For this reason, the antioxidant defense system is crucial in stress conditions [7]. Evidently, oxidative stress is a result of imbalance in the pro- and antioxidant systems with prevalence of prooxidants [9]. However, a prevalence of antioxidants leading to the so-called “antioxidative stress”, as termed by Dündar, may also cause negative effects [10]. Thus, as stressed by Dünbar and Aslan, a delicate balance in pro- and antioxidants is needed for successful defense against oxidative stress [10]. It is noteworthy in this context that adrenoblockers have a significant regulating effect on oxidative processes under stress conditions [11,12]. Since noise-induced metabolic changes are orchestrated by activation of the sympatho-adrenergic signaling system, we sought to investigate the influence of α^2^-adrenoreceptors on noise-induced stress. Two 1,4-benzodioxane derivatives, mesedin and beditin, have been identified as potent α^2^-adrenoblockers with less toxic properties than idazoxan [13]. Hence, we used mesedin and beditin in our study to elucidate the role of α^2^-adrenoreceptors in metabolic and behavioral responses to noise-induced stress. Previous works of our group revealed a strong anti-oxidative effect of beditin in acute noise-induced oxidative damage in rats [11].

As a potent stress factor [14], urban noise can have a long-term impact on cognitive function. Especially, the hippocampus was found to be an important target for stress [15]. Despite the fact that the noradrenergic system’s contribution to anxiety development is well documented [16], the specific role of α^2^-adrenoreceptors in anxiety still needs to be clarified.

Stress is one of the most influential factors for cognitive response impairment [17]. We used a Y-maze to explore the cognitive changes in rats under noise exposure and the influence of beditin and mesedin. The Y-maze is a well-established test for the assessment of spatial memory and exploratory behavior, enabling the measurement of both the quality and quantity of the respective activity [18,19]. Especially in the context of acoustic stress influence on rodent behaviour, it provides sensitive assessment of memory deficits [20].

Our study has revealed concomitant changes in oxidation determined by the level of MDA and α-Tocopherol (α-T) in plasma and erythrocytes’ membrane (EM), as well as cognitive deficits in rats under the noise exposure. These changes were modulated by beditin and mesedin. Our data provides proof for the protective features of both tested α^2^-adrenoblockers. In addition, we identified mesedin to have superior capability of counteracting noise-induced metabolic changes in comparison to beditin. 

## 2. Results 

### 2.1. Biochemical Findings

#### 2.1.1. Changes of MDA Level in Plasma and EM under Chronic Acoustic Stress Conditions and Use of α^2^-Adrenoblockers

**Figure 1:**
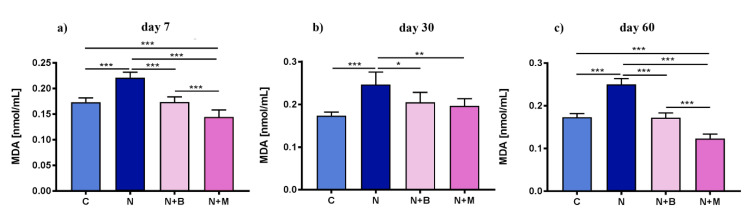
Content of malondialdehyde (MDA) in plasma under acoustic stress conditions after 7, 30, and 60 days. Animals (*n* = 6) were exposed for 60 days to noise (N) or left untreated (C). Mesedin (M) and beditin (B) were injected daily i.p. to the rats exposed to chronic noise (N + M and N + B, respectively). One-way ANOVA with Sidak’s multiple comparison test, * *p* < 0.05, ** *p* < 0.01, *** *p* < 0.001. The mean ± SEM values of plasma content of MDA on days 7, 30, and 60 after exposure of noise and mesedin/beditin are shown in (**a**), (**b**), and (**c**), respectively.

A dramatically increased level of MDA was observed in the noise-treated control after 7, 30, and 60 days in comparison to the untreated control, whereas α^2^-adrenoblockers remarkably diminished the noise impact on plasma MDA content (Figure 1a–c). The effect of mesedine on plasma MDA on days 7 and 60 was superior to that of beditin (*p* < 0.001).

**Figure 2:**
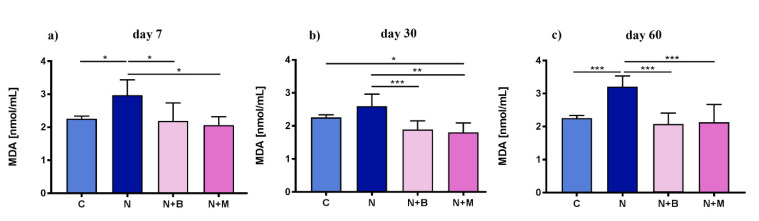
Content of malondialdehyde (MDA) in erythrocytes’ membrane under acoustic stress conditions after 7, 30, and 60 days. Animals (*n* = 6) were treated for 60 days with noise (N) or left untreated (C). Mesedin (M) and beditin (B) were injected daily i.p. to the rats exposed to chronic noise (N + M and N + B, respectively). One-way ANOVA with Sidak’s multiple comparison test, * *p* < 0.05, ** *p* < 0.01, *** *p* < 0.001. The mean ± SEM values of erythrocytes’ membrane content of MDA on days 7, 30, and 60 after exposure of noise and mesedin/beditin are shown in (**a**), (**b**), and (**c**), respectively.

Additionally, in EM, the LPO intensity was significantly increased after 7, 30, and 60 days in the noise-exposed control group. This was shown by comparison of MDA content in untreated vs. noise-treated controls (Figure 2a–c). Administration of α^2^-adrenoblockers led to an MDA decrease, either to the level of untreated control on days 7 and 60 (Figure 2a, b), or even to the values below the naive control (Figure 2c).

#### 2.1.2. Changes of α-T in Plasma and EM under Chronic Acoustic Stress Conditions and Use of α^2^-Adrenoblockers

**Figure 3:**
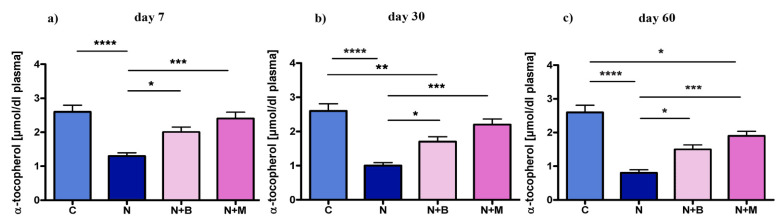
Content of α-T in plasma under chronic acoustic stress conditions. Animals (*n* = 6) were treated for 60 days with noise (N) or left untreated (C). Mesedin (M) and beditin (B) were injected daily i.p. to the rats exposed to chronic noise (N + M and N + B, respectively). One-way ANOVA with Sidak’s multiple comparison test, * *p* < 0.05, ** *p* < 0.01, *** *p* < 0.001, **** *p* > 0.0001. The mean ± SEM values of plasma content of α-T on days 7, 30, and 60 after exposure of noise and mesedin/beditin are shown in (**a**), (**b**) and (**c**) respectively.

As shown in Figure 3, the level of α-T in plasma was significantly lower under chronic acoustic stress conditions at days 7, 30, and 60 in comparison to the untreated control (Figure 3a–c). α^2^-adrenoblockers were capable of alleviating the oxidative response to noise exposure reflected by a higher level of α-T in beditin- and mesedin-treated groups than that of the noise-treated controls (Figure 3a–c).

**Figure 4:**
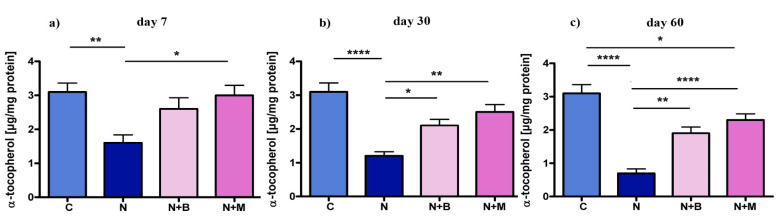
Content of α-T in EM under chronic acoustic stress conditions. Animals (*n* = 6) were treated for 60 days with noise (N) or left untreated (C). Mesedin (M) and beditin (B) were injected daily i.p. to the rats exposed to chronic noise (N + M and N + B, respectively). One-way ANOVA with Sidak’s multiple comparison test, * *p* < 0.05, ** *p* < 0.01, **** *p* > 0.0001. The mean ± SEM values of EM content of α-T on days 7, 30, and 60 after exposure of noise and mesedin/beditin are shown in (**a**), (**b**), and (**c**)**,** respectively.

Similar to the plasma levels, the content of **α**-T in EM of the noise-treated rats was dramatically down-regulated in comparison to untreated controls (Figure 4a–c). Sustained improvement of α-T content in EM was seen from an increased level of α-T after 30 and 60 days of treatment with both adrenoblockers (Figure 2a–c). On day 7, only mesedin was able to significantly increase the α-T level, whereas the difference between the beditin-treated group vs. the noise control group failed to reach statistical significance.

### 2.2. Behavioural Findings

#### 2.2.1. Quantification of Arm Entries in Y-Maze

**Figure 5:**
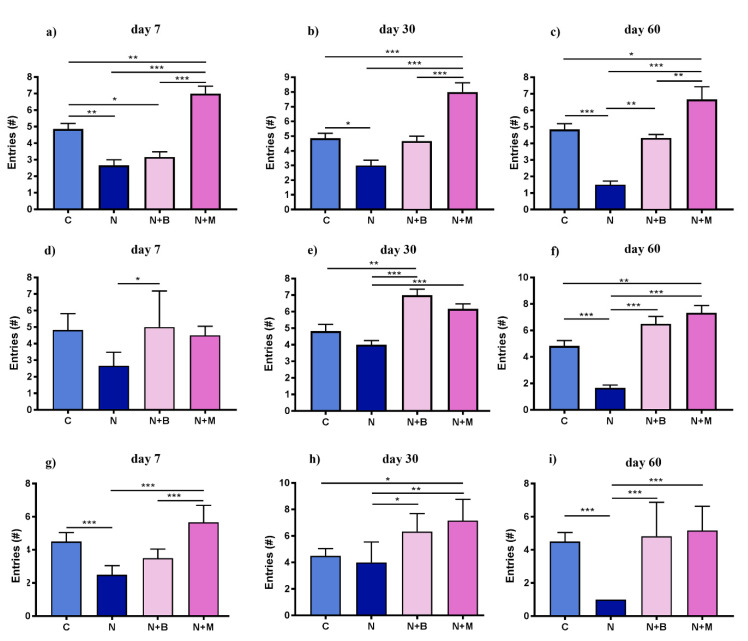
Number of entries to the A–C zones in a Y-maze. Animals (*n* = 6) were treated for 60 days with noise (N) or left untreated (C). Mesedin (M) and beditin (B) were injected daily i.p. to the rats exposed to chronic noise (N + M and N + B, respectively). One-way ANOVA with Sidak’s multiple comparison test, * *p* < 0.05, ** *p* < 0.01, *** *p* < 0.001. The mean ± SEM values of number of entries to the A zone on days 7, 30, and 60 of noise exposure and mesedin/beditin are shown in (**a**), (**b**), and (**c**), respectively. The mean ± SEM values of number of entries to the B zone on days 7, 30, and 60 of noise exposure and mesedin/beditin are shown in (**d**), (**e**), and (**f**), respectively. The mean ± SEM values of number of entries to the C zone on days 7, 30, and 60 of noise exposure and mesedin/beditin are shown in (**g**), (**h**), and (**i**), respectively.

The analysis of arm entries by ANY-maze as a measurement of locomotor activity demonstrated that the noise-treated controls had a smaller number of entries to the A, B, and C arms compared to untreated controls and beditin-/mesedin-treated animals after 7, 30, and 60 days of noise and drug exposure (Figure 5). Notably, mesedin elevated the number of entries to the A arm on days 7, 30, and 60 and to the B arm on day 60 above the level of untreated controls. The highest efficacy of mesedin regarding increase of entries was seen in arm A on all tested days and in C arm entries on day 60. In contrast to mesedin, the difference in entries between the noise and noise + beditin group did not reach a level of significance on day 7 in zone C, as well as on days 7 and 30 in zone A.

#### 2.2.2. Spontaneous Alternations

**Figure 6:**
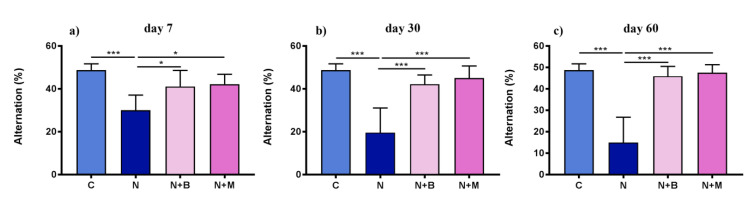
Percentage of alternation in Y-maze. Animals (*n* = 6) were treated for 60 days with (N) noise or left untreated (C). Mesedin (M) and beditin (B) were injected daily i.p. to the rats exposed to chronic noise (N + M and N + B, respectively). One-way ANOVA with Sidak’s multiple comparison test, * *p* < 0.05, *** *p* < 0.001. The mean ± SEM values of percentage of alternations on days 7, 30, and 60 of noise exposure and mesedin/beditin are shown in (**a**), (**b**), and (**c**), respectively.

The alternation percentage as a measure of explorative behavior and working memory revealed a significant impairment of alternation upon noise exposure in comparison to untreated controls (Figure 6). Considering 22.22% alternation to be normal (as explained in the methods part), all experimental animals have performed a higher alternation on day 7. However, while the alternation of mesedin- and beditin-treated groups was remarkably higher on all tested days and already reached the level of untreated controls on day 7, the alternation percentage in noise-treated groups showed an opposite tendency, barely or not reaching the threshold level of 22% on days 30 and 60. In contrast to other parameters shown in this study, both adrenoblockers demonstrated an equal potency of improving spontaneous alternations on all tested days (Figure 6a–c).

#### 2.2.3. Total Mobile Episodes

**Figure 7:**
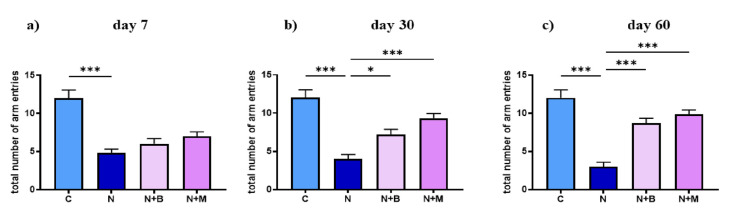
Total number of entries in Y-maze. Animals (*n* = 6) were treated for 60 days with noise (N) or left untreated (C). Mesedin (M) and beditin (B) were injected daily i.p. to the rats exposed to chronic noise (N + M and N + B, respectively). One-way ANOVA with Sidak’s multiple comparison test, * *p* < 0.05, *** *p* < 0.001. Total number of entries reflecting the mobile episodes on days 7, 30, and 60 of noise exposure and mesedin/beditin are shown in (**a**), (**b**), and (**c**), respectively. Data are shown as mean ± SEM.

Assessment of total mobility in Y-maze as a measurement of behavioral activity showed that chronic acoustic stress generally lowered mobility, which was restored by chronic injection of α^2^-adrenoblockers (Figure 7a–c). Our data demonstrated that the noise-treated controls had less arm entries and impaired spatial orientation, accompanied by an increase in the duration of immobility after 7, 30, and 60 days of noise impact compared to other groups (untreated or drug-treated). The pattern was different in beditin-/mesedin-treated animals, where anxiety-induced immobility was less and resulted in more distance traveled in respective groups.

## 3. Discussion

This study demonstrates a clear impact of chronic noise on the level of MDA and α-T in rat plasma and EM. Furthermore, our results indicate that the stress-induced significant changes in biochemical parameters of oxidative stress and cognitive function can be alleviated by α^2^-adrenoblockers, hinting at a strong involvement of α^2^-adrenoceptors in oxidative and behavioral responses to chronic noise.

The noise-induced damage of different organs and systems are found to be mediated by extra formation of ROS [21,22], leading, for instance, to cardiovascular effects (tachycardia, hypertension, etc.). ROS are generated by mitochondria, cytochrome p450, nitric oxide synthase (NOS), etc., which all are attributable to inflammation [23,24]. Increased rate of mitochondrial oxidative processes creates more radicals, capable of damaging such organic molecules as lipids, proteins, and nucleic acids [25]. In this regard, the biological membranes become the most targeted cellular structures, where oxidative stress can be the result of either increased pro-oxidants formation, or the decreased antioxidants’ protective function [26]. The stress-induced age-related disorders, such as arthritis, diabetes, dementia, cancer, atherosclerosis, vascular diseases, obesity, osteoporosis, and metabolic syndromes [27], are mediated by ROS accumulation in the biological systems. In consequence, ROS modulates a big variety of functions and activities such as cell survival, stress-reaction, immune response, as well as aging [25]. Because of high reactivity, the excess of ROS can drive oxidative stress and bring an imbalance to antioxidant and prooxidant ratio [24]. The recent research data testify that natural compounds, such as fatty acids and polyphenols, can reduce oxidative stress and improve immune function [28].

The dysregulation of free radical production leads to changes in the level of oxidation, which is consistent with a shift in MDA level, one of the most common biomarkers of LPO. The enhanced level of MDA is also indicative for abnormality in antioxidant defense. Our results have shown that chronic noise increased the level of MDA in the plasma of the noise-treated control. Strikingly, we observed remarkable restorative effects of beditin and mesedin on the levels of MDA in noise-exposed animals with the most prominent effects obtained upon mesedin administration on days 7 and 60 of chronic noise and drug exposure.

Indicators of oxidative stress can be detected in various body fluids or tissues [29]. Of such indicators, MDA is a product of lipid peroxidation, which immediately reacts with biomolecules in the cells [30]. Its increase indicates intensified oxidative stress in erythrocyte membranes due to an excessive production of ROS by noise action.

Our data demonstrates pronounced deviations in MDA level in the EM of noise-exposed rats. Interestingly, stress hormones such as adrenaline, noradrenaline, cortisol [31,32] lead to structural transitions in EM proteins, along with the deformation of erythrocytes. Structural changes of EM can promote oxidation of proteins and phospholipids targeted by ROS. Notably, stress hormones alter the oxygen transport properties of erythrocytes [33]. An increased level of MDA followed by the development of oxidative stress has been proposed to be one of the pathophysiologic mechanisms of different neurodegenerative diseases including Alzheimer’s [34,35].

As a therapeutic intervention to prevent or even reduce behavioral deficits and the extent of oxidative damage from chronic noise exposure, we employed two novel α^2^-adrenoblockers, mesedin and beditin. In our study, a successful reduction of the noise impact by both α^2^-adrenoblockers evidenced by reduced levels of MDA in the EM was observed. In addition to the MDA-lowering effect, both substances are capable of regulating cholesterol and the intensity of lipid peroxidation as well as membrane phosphoinositides content upon acoustic stress conditions [11,20].

Oxidative stress is characterized by an increase of free radicals, which affect the molecular components of the cell membrane, thereby disturbing their biophysical properties. Transcriptional activities and particularly the cellular signaling pathways are highly dependent on the redox imbalance between the pro- and antioxidants’ levels [36]. Several defenses to protect against the oxidative stress include classical antioxidant enzymes catalase, glutathione peroxidase, and superoxide dismutase as well as non-enzymatic ROS scavengers, such as β-carotene, vitamin C, and vitamin E [37]. Of those antioxidants, the most important ones, Vitamins C and E, cannot be synthesized in the human body [38]. A significance of tocotrienols in defense against oxidative stress leading to inflammation has been demonstrated in decreased arthritis scores, interleukin-1β and H_2_O_2_ levels, and reduced thermal hyperalgesia [39]. Necessity of Vit. E (α-T) for the cellular defense with its widely recognized antioxidant action of scavenging free radicals from the cell membranes, and protecting primarily poly unsaturated fatty acids (PUFA) highly susceptible to oxidative attack [40,41], was shown by us upon chronic acoustic stress condition in rats [42]. 

Here, we show that α-T level was reduced in the plasma and EM of rats with noise exposure (group 2) after 7 days compared to naive control rats. The oxidative damage was continued by chronic exposure of the rats to noise (after 30 and 60 days), which was expressed by dramatically decreased levels of α-tocopherol in the noise-treated control group. Based on our data, it can be suggested that exposure to noise decreased endogenous antioxidant defense due to gradually reduced scavenging of free radicals, leading to cell damage. Our data on beditin and mesedin show that oxidative stress was diminished by both α^2^-adrenoblockers, which is reflected in an increase of α-T in the test groups treated with beditin/mesedin (groups 3 and 4). Thus, administration of α^2^-adrenoblockers can lead to protection of PUFA in cells from LPO membranes via an increase in α-T [43].

Strikingly, mesedin was capable of restoring the investigated parameters to the level of the untreated control group, implying its higher efficacy in comparison to beditin.

Our previous investigations have shown that chronic noise increases carbonylation of plasma proteins and fibrinogen [44], as well as EM proteins [45], which are also known markers for oxidative stress. Administration of α^2^-adrenoblockers mesedin and beditin to the animals under the chronic acoustic stress conditions revealed a regulatory effect on the studied parameters, which was more prominent upon administration of mesedin [44,45]. 

Based on the present results regarding the influence of α^2^-adrenoblockers, it can be concluded that mesedin and beditin possess regulating properties on the MDA content of plasma and EM. Particularly mesedin revealed a pronounced reduction of plasma and EM content of MDA.

Radical scavenging activity of adrenaline and noradrenaline has been suggested to be efficient for reducing oxidative stress. A loss of their activity was also observed during several diseases including depression, sleep disorder, and migraine headache [46,47]. Interestingly, adrenaline and noradrenaline can be both protectors and molecular targets of oxidative stress. It should be also noticed that under proper conditions, both catecholamines can be regenerated to their original form [48]. According to our previous data, one of the main advantages of α^2^-adrenoblocker action is its regulatory effect on lipoprotein metabolism [20]. The major physiological effect of α^2^-adrenoceptors is to block the presynaptic feedback of neurotransmitter release from noradrenergic terminals [49]. Environmental noise exercises its stress-inducing effects through the autonomic nervous system [50] and various connections to cerebral centers responsible for the principal physiological and behavioral effects of the living organism [51]. 

Exposure of laboratory animals to noise induces abnormal behavior, reflected by suppressed exploratory attempts and impaired memory. Some forms of chronic stress could also produce depressive-like symptoms such as anxiety [45]. The hippocampus is an important structure of the central nervous system (CNS), involved in cognitive processes, as well as in the pathogenesis of mood and anxiety disorders through its dorsoventral axis [52,53]. Recent studies suggest that the manipulation of the ventral hippocampus function can directly impact anxiety-related behavior [54]. 

One of the features of the CNS is its high sensitivity to LPO due to high oxygen supply and rich content of polyunsaturated fatty acids. According to Marchasson et al., high plasma level of MDA has been observed in Alzheimer’s disease patients [28]. Noise as a stressor has its negative effect on the whole organism provided by the immediate functional connection of inner ear with the “flight and fight” reaction of animals, which is fulfilled through the autonomous neural system [54]. Beyond this direct pathway, there are also multiple connections of inner ear with the cerebral structures responsible for the processing of emotional and behavioral responses. The cognitive and motor changes in this regard are accompanied by synthesis of sympathetic nerve system’s catecholamines [55]. In this context, the α^2^-adrenoceptors are responsible for the presynaptic inhibition of neurotransmitter release from the noradrenergic nerve terminals [49], as well as for some postsynaptic effects [56]. Different intracellular signaling pathways have been identified by which G protein-coupled receptors can inhibit transmitter release [57].

I-Hsun and colleagues have shown that noise-induced serotoninergic fiber loss occurs in multiple regions of the brain including midbrain, thalamus, hypothalamus, striatum, auditory cortex, and frontal cortex [57]. The high-pressure liquid chromatographic estimation of norepinephrine, epinephrine, dopamine, and serotonin in above-mentioned regions of the rat brain indicates that noise stress can alter the brain biogenic amines content after stress [58]. It is noteworthy that norepinephrine, rather than serotonin, directly activates self-renewing of the precursor cell pool in the adult hippocampus. The norepinephrine-dependent activation of the hippocampal precursors both in vitro and in vivo is associated with the β3-adrenergic receptors activation via adenylate cyclase and cAMP-dependent phosphorylation [59].

The fact that the hippocampus is affected by noise is well documented [16,18]. Many pathological processes, such as post-traumatic stress disorders, involve the hippocampus as the primary damage site. Changes in the neurotransmitters’ contents in the brain are also associated with degenerative diseases of CNS, brain injury, and cognitive disorders [60]. Our results show that chronic noise negatively affects the explorative behavior/spatial memory and the locomotor activity of test animals. For new environment accommodation in Y-maze, the rats generally prefer to enter a new arm of the maze more often rather than returning to a previously visited arm. This test is widely used for an assessment of memory deficits and explorative behavior of rodents [61]. The dramatically reduced alternation percentage of noise-treated test animals in our study can at least partially be ascribed to a decreased behavioral activity as a result of noise-induced stress. One of the reasonable explanations for memory deficits and reduced locomotor activity caused by noise is a high level of anxiety. In our study, a significant increase in the level of anxiety of noise-exposed was observed, which was manifested by freezing behavior and a long stay in one arm of the Y-maze. Our findings suggest that noise-induced stress may lead to spatial memory deficit, which in turn causes orientation impairment [44,45]. Via Y-maze assessment, it was observed that mesedin and beditin treatment of noise-exposed rats resulted in a remarkable and sustained increase in mobility, in memory improvement, and possibly in reduced anxiety. The protective effects of mesedin shown here are in line with our previous data showing neuroprotection provided by mesedin to the hypoxia and glutamate-exposed CNS cells in vitro [62,63]. In addition, the developing of the new therapeutics aimed at regulation of monoamines in the brain is very important in case of neurodegenerative disorders with cognitive impairment including Alzheimer’s disease [64]. 

The noise-induced stress can mimic anxiety-related behaviors by triggering the release of norepinephrine in the central and peripheral nervous systems. With an open field test study, we have previously shown increased anxiety levels induced by noise [44,45]. Chronic administration of α-adrenoblockers led to the inhibition of α-receptor activity and to the reduction of stress-related anxiety levels in rats [44,45].

The anxiety was accompanied by immobility and disturbed orientation of rats. Thus, the noise-associated psycho-emotional stress is capable to mitigate anxiety-related behavior by enhancing the release of norepinephrine in the CNS.

## 4. Material and Methods

### 4.1. Animals

All experiments have been carried out on albino male rats. The animals were bred in the animal facility of Yerevan State Medical University, maintained on a 12 h light/dark cycle with food and water ad libitum. The protocol was approved by the Institutional Animal Care and Ethics Committee of Yerevan State Medical University in accordance with the European Communities Council Directive (86/609/EEC) on care and use of animals for experimental procedures (N8-6/19, 21 March 2019). The rats were accommodated to our laboratory conditions for 7 days prior to the experiment. For the purposes of this study, the rats were assigned to 4 groups (*n* = 6 per group): (1) naive control group; (2) noise-exposed group; (3) noise-exposed and beditin-treated group; (4) noise-exposed and mesedin-treated group. All noise exposed groups were treated with a noise exposure level of 91 dBA and a frequency of 10–20 kHz, produced by a white-noise generator. The animals were exposed to noise for 60 days with a daily exposure of 8 h (from 1:00 a.m. to 9:00 a.m.) and in accordance with established parameters of acoustic stress in rats [65]. Monitoring with a sound meter (ST 11 D) confirmed that the noise was evenly distributed inside the cage. During the entire period of 60 days, the animals of group 3 received daily intraperitoneal injection (i.p.) of beditin (2-amino-4-thiozolyl-1,4-benzodioxan) (2 mg/kg), those of group 4 mesedin (2-(2-methylamino-4-thiozolyl)-1,4-benzodioxan hydrochloride) (10 mg/kg). The first injection was carried out 12 h prior to noise exposure. 

### 4.2. Purification of Erythrocyte Membrane 

Blood samples were taken on days 7, 30, and 60 by cardiopuncture using heparinized syringes under Ketamine anesthesia (0.5–0.75 mg/kg i.p.). Erythrocytes were obtained from the whole blood by washing it four times with phosphate buffered saline, pH 8.0, and through low-speed centrifugation, 1500× *g* for 10 min. Erythrocyte membrane fractions were isolated by osmotic lysis of the washed erythrocytes using lysis solution (5 mM phosphate buffer, pH 8.0, 1 mM EDTA) followed by high-speed centrifugation, 24,000× *g* for 10 min at −4 °C [66]. This process was repeated more than 3 times and hemoglobin was removed with supernatant each time.

### 4.3. Determination of MDA

The MDA level in plasma and erythrocytes membrane was measured according to the method by Esterbauer and Cheeseman [67], based on its reaction with thiobarbituric acid (TBA) at 90–100 °C and measurement of the absorbance at 532 nm. MDA reacts with TBA and produces a pink pigment, which shows maximum absorption at 532 nm. The value of each sample was obtained using a standard curve and expressed as nmol/mL.

### 4.4. Determination of α-T Content in Blood Plasma and EM 

α-T in erythrocyte membranes and blood plasma was determined by the Duggan fluorometric method [68] at an excitation maximum of 295 nm and an emission maximum of 330 nm. Blood plasma (0.75 mL), or 1.5 mL, of erythrocyte membrane suspension were poured into a test tube with a thin section of 15 mL. The samples were diluted with bidistilled water to build a final volume of 5 mL. After vortexing, 6 mL of hexane were added. Then, the tubes were sealed with stoppers and thoroughly shaken for 10 min. α-T was determined after separation in hexane phase. For this purpose, 1 mL of the hexane phase was mixed with 3 mL of absolute alcohol and the sample was subjected to fluorimetry at 295/330 nm. The amount of α-T was calculated using a standard calibration curve and expressed in µmol per dl of plasma and in µg per mg of erythrocyte membrane protein.

### 4.5. Y-Maze

Spontaneous alternation test was used to measure the locomotor activity and spatial memory of tested animals. This was conducted in a Y-shaped maze with three wooden arms of equal size at a 120° angle each. After introduction to the center of the maze, the animal was allowed to freely explore the three arms [69]. An entry was considered when all four limbs were in the respective arm. Alternation was defined as a consecutive entry in three different arms. The sequences ABC, CBA, ACB, or in any order, except for AAC and BCB, were considered as alternation.

Rats were group-housed in standard polycarbonate cages. Approximately 3 min before each assay, the animal was removed from its home cage and placed in a clean holding cage for transfer. Thereafter, it was placed in the center of the Y-maze and allowed to explore the apparatus for 5 min. Each trial was recorded for later analysis, using a video camcorder positioned 2.1 m above the apparatus. After the 5-min test, the rats were taken back to their home cages and the Y-maze was cleaned with 70% ethanol and permitted to dry between the tests. The percentage of alternation was compared within the groups and calculated with the following formula: ((number of alternations)/(total number of arm entries—2) × 100). According to the mathematically calculated probability of entries, the chance level of alternation in Y-maze is considered 22.22%, as suggested by Mao and colleagues [70]. The behavioral parameters were assessed with the help of the ANY-maze behavioral tracking software.

### 4.6. Statistical Analysis

Statistical analyses were performed using GraphPad Prism 8.0 (GraphPad Software, Inc., San Diego, CA, USA). All data is represented as mean ± SEM. For multiple comparisons, one-way ANOVA with Sidak’s test was used. The statistical significance level was set at *p* < 0.05.

## 5. Conclusions

MDA measurement is crucial for the assessment of various pathological states and the effects of pollutants such as environmental noise. Our results demonstrate an increased level of MDA in plasma and EM of animals subjected to chronic noise. These animals have developed an imbalance in the redox status, which favors the oxidation of proteins and phospholipids and the generation of ROS. Dependent on the stress duration, environmental noise can lead to a deficit in spatial memory in rats and may change the functional activity of their membrane surface proteins and signaling complexes. The α^2^-adrenoblockers mesedin and beditin provided a modulatory effect on the investigated parameters under chronic acoustic stress, with most prominent effects shown upon mesedin administration. Summarizing our data, it can be suggested that adrenergic alpha-2 receptor antagonists possess potent antioxidant properties that prevent the extent of LPO, while restoring the level of α-tocopherol in blood plasma and EM under chronic noise conditions.

## Data Availability

Data can be made available by the corresponding author upon reasonable request.
